# High Immunohistochemical Expression of Runt-Related Transcription Factor 2 (RUNX2) Is Associated With High Tumor Grade, Muscle Invasion, Lymph Node Metastasis, and Advanced Stage in Urinary Bladder Cancer

**DOI:** 10.7759/cureus.106976

**Published:** 2026-04-13

**Authors:** Siddharth Garg, Jai K Chaurasia, Devashish Kaushal, E Jayashankar, Shakti K Yadav, Deepti Joshi, Vaishali Walke

**Affiliations:** 1 Pathology and Laboratory Medicine, All India Institute of Medical Sciences, Bhopal, Bhopal, IND; 2 Urology, All India Institute of Medical Sciences, Bhopal, Bhopal, IND

**Keywords:** association, bladder, immunohistochemical, runx2, urinary

## Abstract

Introduction

Urinary bladder cancer is a common malignancy worldwide and remains a significant contributor to cancer-related morbidity and mortality. The recent shift towards personalized oncology has led to the investigation and adoption of various biomarkers as therapeutic targets. However, validated biomarkers predicting tumor behavior and progression in bladder cancer remain limited. The Runt-related transcription factor (RUNX) proteins are transcription factors playing key roles in signaling pathways associated with cancer development. There are limited studies in the literature addressing the role of RUNX2 in urinary bladder cancers. Therefore, we have attempted to analyze the immunohistochemical expression of RUNX2 in urinary bladder cancer in this study and to evaluate the significance of its immunoexpression in relation to clinicopathological parameters such as age and gender of patient, tumor size and site, tumor grade, histological subtype, muscle invasion, lymph node metastasis, and pTNM stage.

Materials and methods

This observational study was carried out in the Pathology department of our institute. Sixty histopathologically confirmed consecutive cases of urinary bladder carcinoma over a period from 1st January 2022 to 31st March 2025 were included. Immunohistochemistry was performed on these cases using archival paraffin blocks and the RUNX2 antibody. The final immunohistochemical results were analyzed in relation to clinicopathological variables.

Result

The data were analyzed using SPSS (version 23.0, IBM Corp., Armonk, New York, USA). To assess statistical significance (p-value) and correlations, the chi-square test and Fisher's exact test were used. For establishing statistical significance, a p-value of less than 0.05 was considered. High immunohistochemical expression of RUNX2 exhibited a significant association (p < 0.05) with high tumor grade, muscle invasion, lymph node metastasis, and advanced stage (pTNM) of the tumor.

Conclusions

This study documents that there exists a statistically significant association between immunoexpression of RUNX2 and grade of tumor, muscle invasion, advanced stage, and lymph node metastasis in urinary bladder cancers, which can guide further management and treatment and can be utilized in the near future for the development of targeted therapy for urinary bladder cancer patients.

## Introduction

Urinary bladder cancer represents a significant global health burden and continues to rank among the most commonly diagnosed malignancies worldwide [1]. Clinically, it is broadly categorized into non-muscle-invasive bladder cancer (NMIBC) and muscle-invasive bladder cancer (MIBC), two entities that differ markedly in their biological behavior, molecular profile, and therapeutic approach. NMIBC is generally managed with transurethral resection of bladder tumor (TURBT) followed by risk-adapted intravesical therapy, whereas MIBC typically necessitates a more aggressive strategy including neoadjuvant chemotherapy and radical cystectomy with lymph node dissection and urinary diversion [2,3].

The heterogeneity in disease progression and clinical outcomes has driven increased interest in identifying reliable biomarkers to aid prognostication and guide personalized therapeutic strategies. Despite ongoing research, the availability of robust biomarkers capable of accurately predicting tumor behavior, progression, and response to therapy in bladder cancer remains limited [4].

The Runt-related transcription factor (RUNX) family comprises key regulators of gene expression involved in cellular differentiation, proliferation, and oncogenic transformation [5]. Among these, RUNX2 was initially characterized for its pivotal role in osteoblast differentiation and skeletal development. However, emerging evidence has highlighted its broader involvement in tumor biology across various malignancies [6,7]. RUNX2 has been implicated in multiple oncogenic processes, including tumor initiation, proliferation, metastasis, angiogenesis, and resistance to therapy.

In the context of urinary bladder cancer, RUNX2 is believed to contribute to tumor progression through several molecular mechanisms. These include enhancement of glutamine metabolism, promotion of epithelial-mesenchymal transition (EMT), and facilitation of tumor cell invasion via modulation of extracellular matrix components and cell adhesion molecules. Additionally, its interaction with cancer-associated fibroblasts and the tumor microenvironment further supports tumor aggressiveness and disease progression [7-12]. RUNX2 has also been associated with increased cellular proliferation and reduced apoptotic activity, thereby contributing to tumor growth and invasive potential.

Data regarding the expression and clinicopathological significance of RUNX2 in bladder cancer from the Indian population are limited. The objective of the study was to evaluate the immunohistochemical expression of RUNX2 in urinary bladder carcinoma and to assess its association with clinicopathological variables, including patient’s age and gender, tumor site, tumor grade, histological subtype, muscle invasion, lymph node metastasis, and pathological tumor stage (pTNM).

## Materials and methods

This observational study was conducted in the Pathology department of our institute, with approval from the Institutional Human Ethics Committee (IHEC-SR-AIIMS/BPL/IECSR/JAN/23/PG/10). Sixty histopathologically confirmed consecutive cases of urinary bladder carcinoma encountered from 1st January 2022 to 31st March 2025 were included. Out of the total 60 cases, 42 (70%) specimens were of transurethral resection of bladder tumor (TURBT), and the remaining 18 (30%) specimens were resected cystectomy specimens. The clinical details of the patients were retrieved from the archival records. The histopathology of the tumor, including tumor size and site, and pTNM stage (AJCC 8th edition), was noted [13]. The hematoxylin and eosin (H&E) stained slides of selected cases were retrieved and examined for histological subtype, tumor grade, and lymph node metastasis. The immunohistochemistry (IHC) for RUNX2 was then performed on sections obtained from selected paraffin-embedded blocks.

Inclusion criteria

Urinary bladder carcinoma cases with histopathological confirmation were included.

Exclusion criteria

Blocks with inadequate diagnostic tissue material were excluded.

Immunohistochemistry

For performing immunohistochemistry (IHC), first, paraffin-embedded blocks fulfilling the inclusion criteria were retrieved from the archives. Using microtomy on the paraffin-embedded blocks of selected cases, 3-5 micron-thick sections were obtained on charged slides. Immunohistochemistry (IHC) was then performed using the commercially available rabbit polyclonal RUNX2 antibody (Clone: Ab-AF5186#1486, pack size 100 µl, concentration of 1 mg/ml) from Affinity Biosciences, Cincinnati, OH, USA. The IHC was performed after antibody dilution (1:100), using an automated immunohistochemistry stainer. Known histopathologically confirmed cases of carcinoma of the urinary bladder were selected for RUNX2 as positive controls. For negative controls, cases diagnosed as cystitis and benign urothelial tumors such as papillomas were used.

Interpretation of immunohistochemistry

Positive immunohistochemical expression of RUNX2 was interpreted as the formation of a brown-colored product at the site of the target antigen and was analyzed by assessing the percentage (%) of positive cells and the intensity of nuclear staining. Nuclear staining was taken as positive for RUNX2. The score for staining intensity and the % of positive cells was evaluated (Table 1). An immunohistochemical expression score ranging from 0 to 12 was obtained by multiplying the staining intensity score and % of positive cells. A final score of <4 will be considered as low immunohistochemical expression, and ≥4 will be considered as high immunohistochemical expression (Table 1) [14].

**Table 1:** Immunohistochemical staining score of RUNX2. RUNX2: Runt-related transcription factor 2.

Intensity score				Percentage of positive cells					Final staining score	
Score 0	Score 1	Score 2	Score 3	Score 0	Score 1	Score 2	Score 3	Score 4	Low	High
											Absence of staining	Weak nuclear blush of staining	Moderate nuclear staining	Intense nuclear staining	0%	1-25%	26-50%	51-75%	76-100%	<4	≥4

Statistical analysis

The data were analyzed using the Statistical Package for the Social Sciences (SPSS) statistics software (version 23.0) (IBM Corp., Armonk, New York, USA). For categorical variables, descriptive statistics, frequency analysis, and percentage analysis were used, and for continuous variables, means and standard deviations (SDs) were calculated. Chi-square and Fisher's exact tests were used to assess statistical significance, as appropriate. Fisher's exact test was applied when expected cell counts were less than 5. For establishing statistical significance, a p-value of less than 0.05 was considered.

## Results

Among the 60 cases, a high immunohistochemical expression score of RUNX2 (score ≥4) was observed in 47 (78.3%) cases. A low score of <4 was observed in 13 (21.7%) cases.

Clinicopathological association

The association of immunohistochemical expression of RUNX2 with clinicopathological variables, such as patient’s age and gender, tumor site and size, tumor grade, histological subtype, muscle invasion, lymph node metastasis, and tumor stage (pTNM) was done (AJCC 8th Edition, Tables 2, 3).

**Table 2:** Association of immunohistochemical expression of RUNX2 with various clinicopathological variables. *Fisher's exact test was used when expected cell counts were <5. **Chi-square test was used when expected cell counts were ≥5. RUNX2: Runt-related transcription factor 2.

Clinicopathological variables	No. of cases (%)	Immunohistochemical expression of RUNX2		Fisher's exact test	
		High (%)	Low (%)	χ^2^	p-value
Age groups (in years)					
20-29	2 (3.3)	2 (4.3)	0 (0.0)	14.926	0.016*
30-39	3 (5.0)	3 (6.4)	0 (0.0)		
40-49	4 (6.7)	1 (2.1)	3 (23.1)		
50-59	15 (25.0)	15 (31.9)	0 (0.0)		
60-69	24 (40.0)	18 (38.3)	6 (46.2)		
70-79	10 (16.7)	6 (12.8)	4 (30.8)		
80-89	2 (3.3)	2 (4.3)	0 (0.0)		
Gender					
Male	49 (81.7)	38 (80.9)	11 (84.6)	0.096	1.000*
Female	11 (18.3)	9 (19.1)	2 (15.4)		
Size (in mm)					
<30 mm	23 (38.3)	19 (40.4)	4 (30.8)	0.402	0.749**
≥30 mm	37 (61.7)	28 (59.6)	9 (69.2)		
Tumor site					
Posterior bladder wall	16 (26.7)	8 (17.0)	8 (61.5)	-	0.003*
Tumor grade					
High grade	34 (56.7)	32 (68.1)	2 (15.4)	9.472	0.002**
Low grade	26 (43.3)	15 (31.9)	11 (84.6)		
Histological subtypes					
Papillary urothelial carcinoma (invasive and non-invasive)	42 (70.0)	31 (66.0)	11 (84.6)	2.441	0.836*
Invasive urothelial carcinoma conventional	10 (16.7)	9 (19.1)	1 (7.7)		
Invasive urothelial carcinoma with squamous differentiation	4 (6.7)	3 (6.4)	1 (7.7)		
Clear cell urothelial carcinoma	1 (1.7)	1 (2.1)	0 (0.0)		
Squamous cell carcinoma	1 (1.7)	1 (2.1)	0 (0.0)		
Adenocarcinoma	1 (1.7)	1 (2.1)	0 (0.0)		
Sarcomatoid urothelial carcinoma	1 (1.7)	1 (2.1)	0 (0.0)		

**Table 3:** Association of immunohistochemical expression of RUNX2 with muscle invasion, T stage, and lymph node metastasis. *Fisher's exact test was used when expected cell counts were <5. **Chi-square test was used when expected cell counts were ≥5. RUNX2: Runt-related transcription factor 2.

Clinicopathological variables	No. of cases (%)	Immunohistochemical expression of RUNX2		Fisher's exact test	
		High (%)	Low (%)	χ^2^	p-value
Muscle invasion					
Present	31 (51.7)	29 (61.7)	2 (15.4)	8.748	0.003**
Absent	29 (48.3)	18 (38.3)	11 (84.6)		
T stage					
Ta	7 (11.7)	4 (8.5)	3 (23.1)	4.069	0.028*
T1	22 (36.7)	15 (31.9)	7 (53.8)		
T2a	15 (25.0)	14 (29.8)	1 (7.7)		
T2b	6 (10.0)	4 (8.5)	2 (15.4)		
T3a	4 (6.7)	4 (8.5)	0 (0.0)		
T3b	2 (3.3)	2 (4.3)	0 (0.0)		
T4a	3 (5.0)	3 (6.4)	0 (0.0)		
Lymph node metastasis (N stage)					
Nx	43 (71.7)	30 (63.8)	13 (100.0)	6.561	0.012*
N0	11 (18.3)	11 (23.4)	0 (0.0)		
N1	3 (5.0)	3 (6.4)	0 (0.0)		
N2	3 (5.0)	3 (6.4)	0 (0.0)		

Age and gender

The patient's age ranged from 23 years to 84 years, with a mean age of 59.60 ± 12.48 years. The association between immunohistochemical expression of RUNX2 and age groups (p = 0.016) was found to be statistically significant (Table 2). Out of the 60 patients included, the majority 49 (81.67%) were males and 11 (18.3%) were females. No statistically significant association was observed between immunohistochemical expression of RUNX2 (p = 1.000) and the gender of the patient (Table 2).

Tumor size and site

Maximum and minimum sizes of the tumor were 120 mm and 5 mm, respectively. Twenty-three (38.3%) cases presented with tumors of size less than <30 mm, and the remaining 37 (61.7%) cases presented with a tumor size of ≥30 mm (Table 2). No statistically significant association was observed between immunohistochemical expression of RUNX2 (p = 0.749) and the size of the tumor (Table 2). The majority of bladder tumors (26.7%) were located on the posterior bladder wall. Statistically significant association was observed between immunohistochemical expression of RUNX2 and tumor site involving the posterior bladder wall (p = 0.003) (Table 2).

Tumor grade

In the present study, the cases were categorized into high- and low-grade [9]. The majority of 34/60 (56.7%) cases belonged to the high-grade category, and 26/60 (43.3%) cases were categorized as low grade. Out of 47 (78.3%) cases showing high immunohistochemical expression of RUNX2 (score ≥ 4), 32 (68.1%) cases were of high tumor grade, while the remaining 15 (31.9%) cases belonged to the low tumor grade category (score < 4) (Table 2). Statistically significant association was observed between immunohistochemical expression of RUNX2 (p = 0.002) with the grade of the tumor (Table 2).

Histological subtype

**Figure 1:**
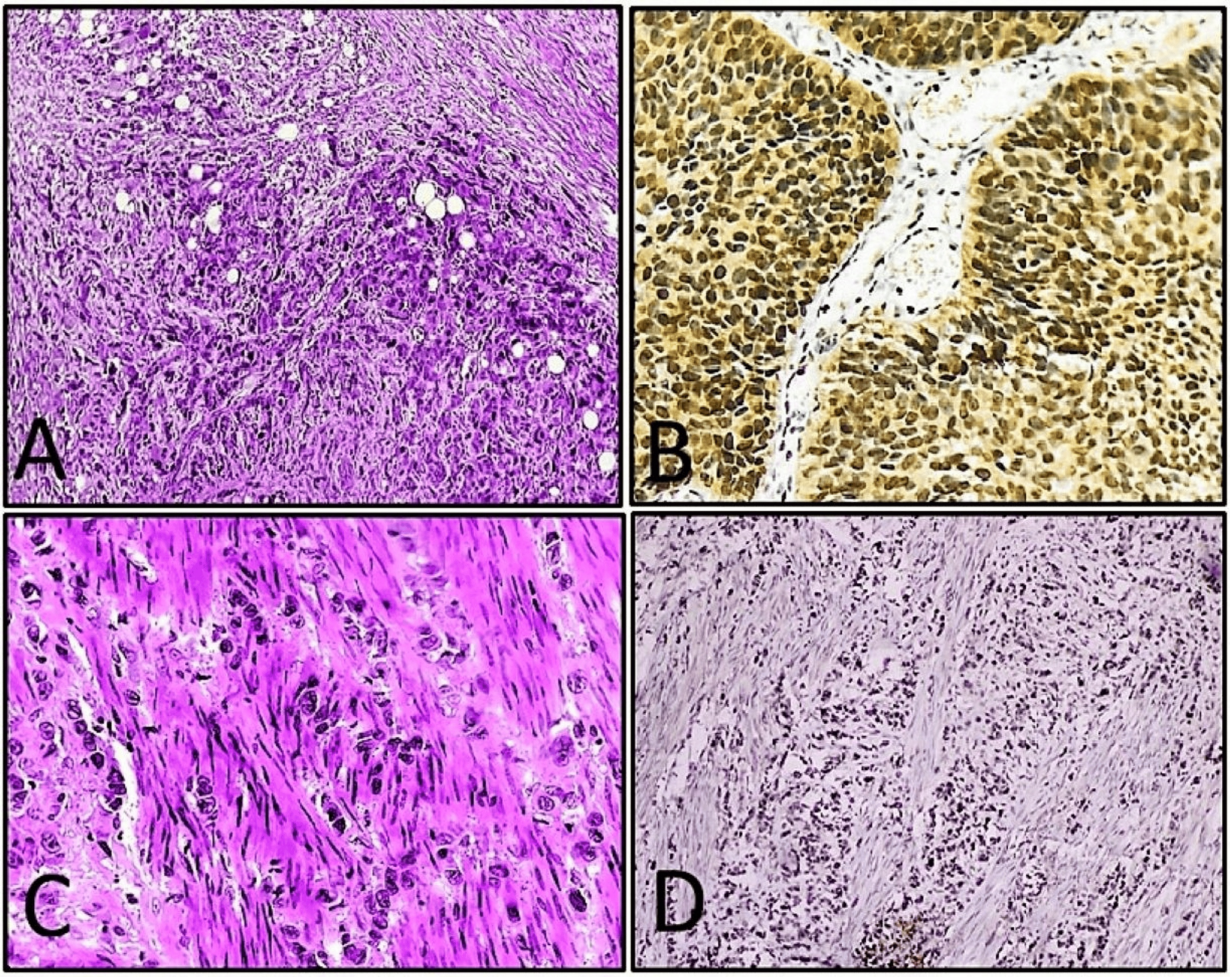
(A) Invasive high-grade urothelial carcinoma (H&E, 10x); (B) high immunohistochemical expression of RUNX2 with strong nuclear positivity (score 12) in the same case of high-grade urothelial carcinoma (IHC RUNX2, 40x); (C) muscle invasion by tumor cells high-grade urothelial carcinoma (H&E, 40x); (D) high immunohistochemical expression of RUNX2 with strong nuclear positivity (score 12) with muscle invasion by tumor cells (IHC RUNX2, 10x). IHC: immunohistochemistry; H&E: hematoxylin and eosin; RUNX2: Runt-related transcription factor 2.

All cases were categorized into low-grade and high-grade by histological examination. Tumors with marked nuclear atypia and loss of architectural polarity, with hyperchromatic nuclei showing marked nuclear pleomorphism, and with the highest-grade component representing >5% of the tumor were categorized as high grade (Figure 1A) [15].

**Figure 2:**
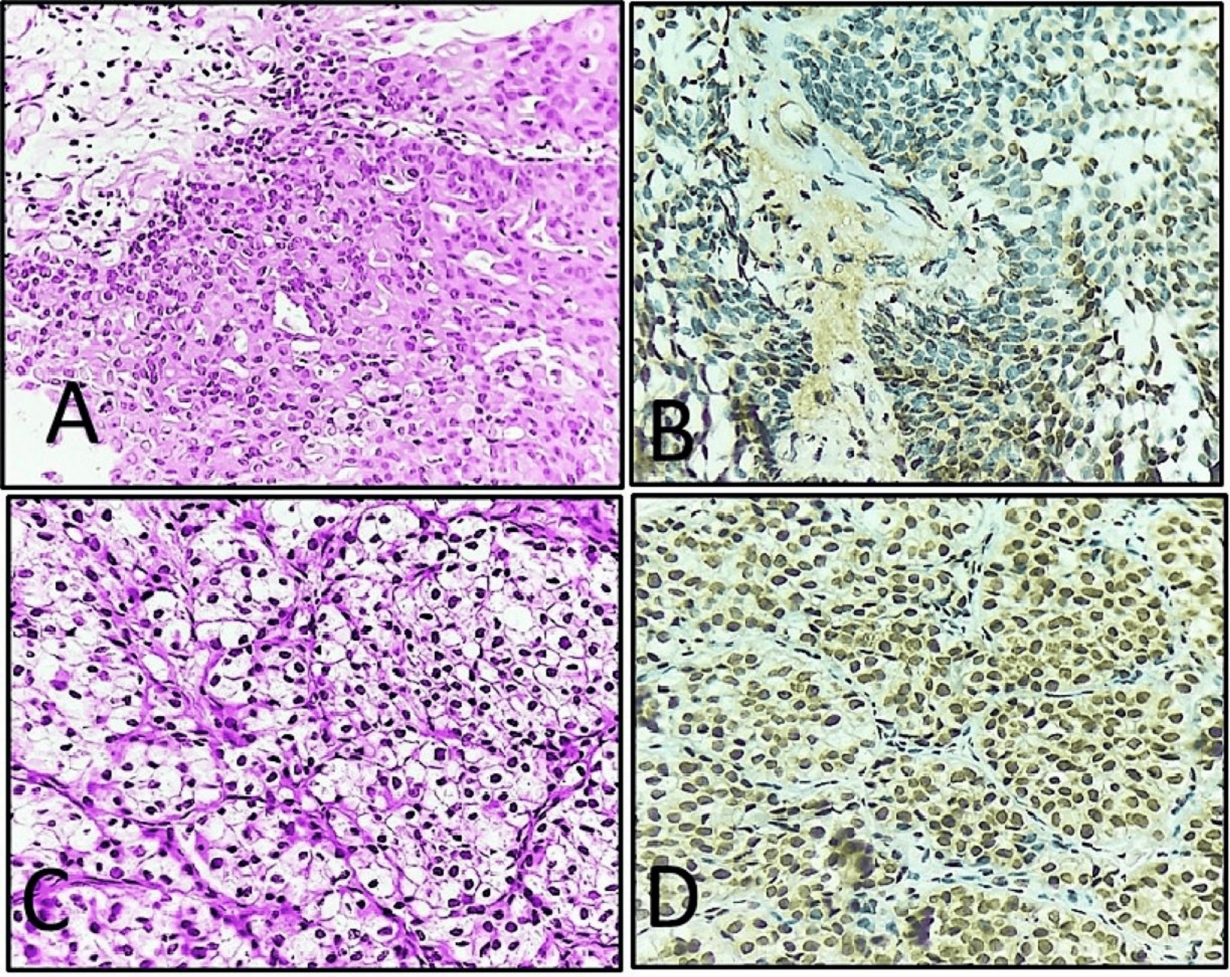
(A) Low-grade urothelial carcinoma (H&E, 40x); (B) low-immunohistochemical expression of RUNX2 with weak nuclear positivity (score 2) in the same case of low-grade urothelial carcinoma (IHC RUNX2, 40x); (C) tumor cells with clear cytoplasm (glycogen-rich) in clear cell urothelial carcinoma (H&E, 40x); (D) strong nuclear positivity (score 12) with high immunohistochemical expression of RUNX2 in same case of clear cell urothelial carcinoma (IHC RUNX2, 40x). IHC: immunohistochemistry; H&E: hematoxylin and eosin; RUNX2: Runt-related transcription factor 2.

Tumors showing mild nuclear atypia and mild distortion of architectural polarity, absence of marked nuclear pleomorphism and hyperchromasia were categorized as low-grade urothelial cancers (Figure 2A).

All special histological subtypes of urinary bladder carcinomas and those showing dedifferentiation were considered high-grade tumors. Following histological types of urothelial carcinomas were included in the study: (1) non-invasive papillary urothelial carcinoma, low grade, (2) non-invasive papillary urothelial carcinoma, high grade, (3) invasive urothelial carcinoma (conventional), (4) urothelial carcinoma with squamous differentiation, (5) squamous cell carcinoma, (6) clear cell urothelial carcinoma, (7) adenocarcinoma, and (8) sarcomatoid urothelial carcinoma.

**Figure 3:**
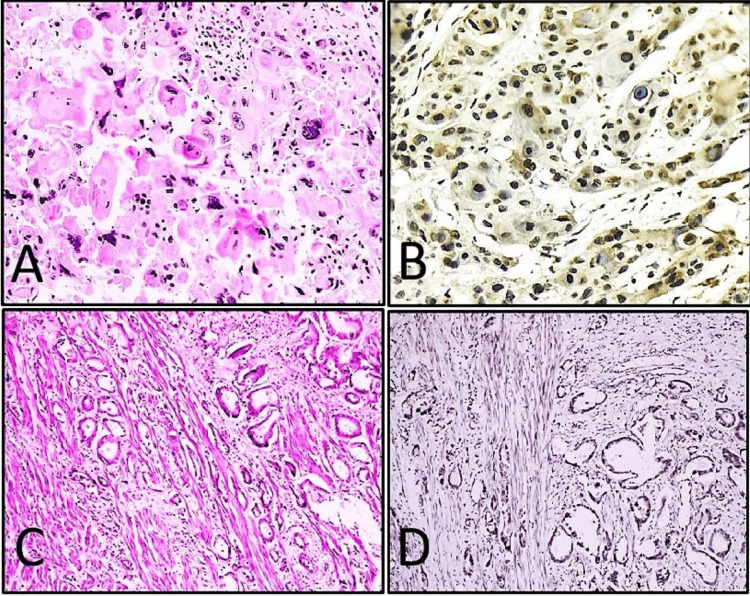
(A) Squamous cell carcinoma with keratin deposition (H&E, 40x); (B) tumor cells with high nuclear immunohistochemical expression of RUNX2 (score 12) in the same case of squamous cell carcinoma (IHC RUNX2, 40x); (C) adenocarcinoma with muscle-invasive glands (H&E, 40x); (D) strong nuclear positivity (score 12) with high immunohistochemical expression of RUNX2 in the same case of urothelial adenocarcinoma (IHC RUNX2, 10x). IHC: immunohistochemistry; H&E: hematoxylin and eosin; RUNX2: Runt-related transcription factor 2.

Out of 60 cases, papillary urothelial carcinoma (42/60, 70%) was the predominant histological subtype, followed by invasive urothelial carcinoma conventional (10/60, 16.7%) (Figure 1A), invasive urothelial carcinoma with squamous differentiation (4/60, 6.7%) and 1 case (1.7%) each of clear cell urothelial carcinoma (Figures 2C, 2D), squamous cell carcinoma (Figures 3A, 3B), adenocarcinoma (Figures 3C, 3D) and sarcomatoid urothelial carcinoma. Histological subtypes of invasive urothelial carcinoma and those with divergent differentiation were all considered high-grade tumors and showed high RUNX2 immunoexpression (Figures 2D, 3B, 3D). However, no statistically significant association was observed between immunohistochemical expression of RUNX2 (p = 0.836) with histological subtype of tumor (Table 2).

Muscle invasion

Muscle-invasive bladder carcinoma (MIBC) was defined as a malignant neoplasm of urothelial origin that demonstrates histological evidence of invasion into the muscularis propria (detrusor muscle) of the urinary bladder wall (Figure 1C). Out of the 60 cases, muscle invasion was present in 31 (51.7%) cases, while the remaining 29 (48.3%) cases showed no evidence of muscle invasion (Table 3). The association between immunohistochemical expression of RUNX2 and muscle invasion was found to be statistically significant (p = 0.003) (Table 3).

Tumor stage

T Stage

The primary tumor T staging was determined using TNM staging for bladder cancer (AJCC 8th Edition, 2017) (Table 3). Out of the 60 cases, 7 (11.7%) were staged as Ta, 22 (36.7%) as T1, 21 (35.0%) as T2, 6 (10.0%) as T3, and 4 (6.7%) as T4 (Table 3). Statistical analysis demonstrated a significant association between immunohistochemical expression of RUNX2 (p = 0.028) with advancing tumor (T) stages, with high RUNX2 immunohistochemical expression more frequently observed in advanced stage muscle invasive tumors (≥T2; invasion into muscularis propria or beyond) compared to early-stage non-muscle invasive tumors (Ta and T1; confined to the urothelium or subepithelial connective tissue) (Table 3).

Lymph Node Metastasis (N Stage)

The nodal staging was determined using TNM Staging for Bladder Cancer (AJCC 8th Edition) (Table 3). Out of 60 cases, nodal metastasis could be assessed in 17 (28.3%) cases. Among the 17 cases, 11 (64.7%) exhibited no lymph node metastasis (N0 stage), while lymph node metastasis was identified in 6 (35.3%) cases, with 3 (50%) cases belonging to each stage N1 and stage N2 (Table 3). The association of high immunohistochemical expression of RUNX2 and nodal metastasis-positive cases at N1 and N2 stages was found to be significant (p = 0.012).

M Stage

In all 60 cases, distant metastasis could not be assessed (Mx stage).

## Discussion

Biomarkers have become increasingly important in understanding tumor biology, predicting disease progression, and identifying potential therapeutic targets in malignancies. Among emerging molecular markers, RUNX2 has gained attention due to its involvement in multiple oncogenic pathways [10-12]. Evaluating its immunohistochemical expression in urinary bladder carcinoma may provide useful insights into tumor behavior and assist in prognostication. However, available literature on RUNX2 expression in bladder cancer, particularly from the Indian population, remains limited.

Out of the 60 cases of urinary bladder cancer, 47 (78.3%) cases showed high immunohistochemical expression of RUNX2. Similarly, Ibrahim and Abdelrahman reported high RUNX2 immunohistochemical expression in 42 cases (67.7%) of urinary bladder carcinoma cases [14]. The relatively high frequency of expression in both studies suggests a consistent association between RUNX2 and urothelial malignancies.

The mean age of patients in the present study was 59.60 ± 12.48 years, with age ranging from 23 years to 84 years. The present study revealed a statistically significant association (p = 0.016) between immunohistochemical expression of RUNX2 and age groups of the patients (Table 2). High RUNX2 immunohistochemical expression was predominantly noted in the age group between 50 and 69 years of age (Table 2). These results indicate that high immunohistochemical expression of RUNX2 significantly associates with middle to older age groups, suggesting potential implications for tumor biology and aggressiveness in these age groups.

In our study, the immunohistochemical expression levels of RUNX2 showed no significant association with gender (p > 0.05) (Table 2), indicating that the immunohistochemical expression of these biomarkers is likely independent of gender-based differences in patients with urinary bladder carcinoma. This observation aligns with a previous study done by Abdelzaher and Kotb, who also reported no significant association between RUNX2 (p = 0.628) and patients' gender [16].

An interesting finding revealed in this study was a positive trend toward an association of high immunohistochemical expression of RUNX2 with larger tumor sizes; however, this did not reach statistical significance (p = 0.749) (Table 2). Ibrahim and Abdelrahman also reported no statistically significant association (p = 0.374) between immunohistochemical expression of RUNX2 and the size of the tumor, suggesting that tumor size alone may not be a reliable determinant of RUNX2 expression [14].

Another finding in the present study was the significant association between RUNX2 expression and tumor location, particularly involving the posterior bladder wall (p = 0.003) (Table 2). These findings indicate that certain bladder locations, such as the posterior bladder wall, may have distinct biological behavior influencing RUNX2 expression. Hence, larger cohort studies are required to validate these observations and to better define the potential tumor-site-specific roles of RUNX2 in urinary bladder carcinomas.

A strong correlation was identified between RUNX2 expression and tumor grade (p = 0.002), with high-grade tumors showing markedly increased expression (Figures 1A, 1B) compared to low-grade tumors (Figures 2A, 2B) (Table 2), suggesting that elevated RUNX2 expression is closely linked with increased tumor aggressiveness and may serve as a valuable biomarker for grading and prognostication in urinary bladder carcinomas. Similarly, a significant association was also documented by Ibrahim and Abdelrahman (p = 0.003) as well as by Abdelzaher and Kotb (p = 0.017) [14,16]. However, RUNX2 immunoexpression and its association with histological subtype of bladder carcinoma were found to be statistically insignificant (p = 0.836) (Table 2). This suggests that RUNX2 expression may be independent of histological differentiation and more closely related to tumor behavior rather than morphological subtype. To our knowledge, no previous literature has documented an association between immunohistochemical expression of RUNX2 with various histological subtypes of urinary bladder carcinomas.

A significant positive association (p = 0.003) was observed between immunohistochemical expression of RUNX2 and the presence of muscle invasion in urinary bladder carcinomas (Table 3). Among the 47 cases showing high immunohistochemical expression of RUNX2, histologic evidence of muscle invasion was found in 29 cases (61.7%) (Figure 1C), whereas out of the 13 cases showing low immunohistochemical expression of RUNX2, histologic evidence of muscle invasion was noted in only 02 cases (15.4%) (Table 3). These findings strongly suggest that high immunohistochemical expression of RUNX2 is associated with muscle-invasive urinary bladder carcinoma (MIBC) (Figure 1D) and indicative of higher tumor invasiveness and progression. These findings align with Abdelzaher and Kotb's study, who were among the first to report this correlation, documenting that high RUNX2 immunoexpression was significantly linked to muscle-invasive disease [16]. Ibrahim and Abdelrahman also found a strong correlation (p = 0.004) between high RUNX2 immunoexpression and muscle-invasive status [14]. These findings underscore that high immunohistochemical expression of RUNX2 strongly indicates invasive disease, aligning with its roles in tumor aggressiveness, epithelial-mesenchymal transition (EMT), and metastasis.

In this study, a statistically significant positive association (p = 0.028) was observed between immunohistochemical expression of RUNX2 and advancing tumor (T) stage (Table 3). Among early-stage tumors (Ta and T1), 34.5% cases exhibited low immunohistochemical expression of RUNX2, while 65.5% exhibited high immunohistochemical expression of RUNX2 whereas, advanced-stage tumors (≥T2) demonstrated a clear shift toward high immunohistochemical expression of RUNX2, with 90.3% cases showing high immunohistochemical expression and only 9.7% showing low immunohistochemical expression of RUNX2 (Table 3). Similar findings were reported by Ibrahim and Abdelrahman, as well as by Abdelzaher and Kotb, with significant p-values of 0.021 and 0.003, respectively [14,16]. These findings indicate that high immunohistochemical expression of RUNX2 is associated with advancing tumor depth and invasiveness, supporting its potential role as a marker of tumor progression in urinary bladder carcinomas.

The analysis of immunohistochemical expression of RUNX2 in relation to positive lymph node metastasis (N stage) showed a statistically significant association (p = 0.012) (Table 3). These findings are in concordance with a study from Ibrahim and Abdelrahman, who also demonstrated a significant correlation (p = 0.045) [14]. These findings suggest that elevated immunohistochemical expression of RUNX2 may serve as a potential indicator of regional lymph node involvement in urinary bladder carcinoma. Their overexpression could reflect an underlying EMT-driven mechanism facilitating metastatic progression.

Limitations of the study

A relatively small sample size of 60 consecutive cases of urinary bladder carcinoma, collected between 1st January 2022 and 31st March 2025, was included in the study, which may not be fully representative of the broader patient population. Moreover, a multivariate analysis to assess the independent predictive value of RUNX2 expression could not be performed due to the relatively small sample size and limited number of outcome events (particularly for lymph node metastasis), which could have led to unreliable estimates. Furthermore, no formal power calculation was performed due to these reasons. Also, interobserver variability was also not assessed in this study. Therefore, validation of the findings of the study requires larger, multicentric cohorts to establish a statistically strong correlation. Additionally, long-term follow-up of patients was not done in the study, which prevented a comprehensive evaluation of survival outcomes and the prognostic value of these markers.

## Conclusions

This study underscores that there exists a statistically significant association between immunoexpression of RUNX2 and grade of tumor, muscle invasion, advanced stage, and lymph node metastasis in urinary bladder carcinomas. The results of our study can give way to the larger cohort studies in the future to further affirm the prognostic role of RUNX2 immunohistochemical expression in urinary bladder cancer, directing the research on targeted therapy for treatment of urinary bladder cancer, as no specific RUNX2-targeted therapy is currently available. Moreover, immunohistochemistry used in this study can be used as an economic and easily available platform to analyze the RUNX2 immunohistochemical expression.
